# Clinical Implications of Cardiac Hyperpolarized Magnetic Resonance Imaging

**DOI:** 10.1186/1532-429X-15-93

**Published:** 2013-10-08

**Authors:** Oliver J Rider, Damian J Tyler

**Affiliations:** 1 University of Oxford Centre for Clinical Magnetic Resonance Research, Division of Cardiovascular Medicine, Radcliffe Department of Medicine, University of Oxford, Oxford, UK; 2 Oxford Metabolic Imaging Group, University of Oxford, Oxford, UK; 3 Department of Physiology, Anatomy and Genetics, University of Oxford, Parks Road, Oxford, OX1 3PT, UK

**Keywords:** Hyperpolarized, Carbon-13 (^13^C), Pyruvate, Cardiac Metabolism

## Abstract

Alterations in cardiac metabolism are now considered a cause, rather than a result, of cardiac disease. Although magnetic resonance spectroscopy has allowed investigation of myocardial energetics, the inherently low sensitivity of the technique has limited its clinical application in the study of cardiac metabolism. The development of a novel hyperpolarization technique, based on the process of dynamic nuclear polarization, when combined with the metabolic tracers [1-^13^C] and [2-^13^C] pyruvate, has resulted in significant advances in the understanding of real time myocardial metabolism in the normal and diseased heart *in vivo.* This review focuses on the changes in myocardial substrate selection and downstream metabolism of hyperpolarized ^13^C labelled pyruvate that have been shown in diabetes, ischaemic heart disease, cardiac hypertrophy and heart failure in animal models of disease and how these could translate into clinical practice with the advent of clinical grade hyperpolarizer systems.

## Introduction

It is now widely accepted that cardiac substrate utilisation is altered in many cardiac diseases [1,2] and that this is likely to alter myocardial ATP production and, as a consequence, cardiac function [3-5]. As a result, changes in cardiac metabolic substrate utilization are now being considered as a cause, rather than a consequence, of cardiac disease [5]. It is also anticipated that better understanding of these metabolic changes will lead to novel therapeutic targets to treat a wide variety of cardiac diseases.

However, despite this clear potential for metabolic therapies to treat heart disease, current treatments based on altering substrate selection have only had limited success [6-8]. This is, at least in part, due to the fact that controversy remains over the exact nature of metabolic alterations. When coupled with a poor understanding of the mechanisms underlying these changes, this makes targeted pharmacological therapy difficult to achieve. This is further hampered by the fact that the majority of metabolic investigations are either carried out using destructive *ex vivo* methods, which disturb the regulation of metabolism, or using *in vivo* radiolabeled tracer techniques (Positron Emission Tomography, PET and Single Photon Emission Computed Tomography, SPECT) that cannot distinguish between the tracer and its metabolic products [9].

Magnetic resonance spectroscopy (MRS) is an ideal tool for the non-invasive study of metabolism, due to the extensive range of compounds it can detect, using nuclei such as carbon (^13^C) and phosphorus (^31^P), and it has been used many times to interrogate cardiac energy metabolism in animals and in patients [10-12]. However, applications of MR measurements of metabolism have been limited by an intrinsically low sensitivity. In standard MRI, the high proton concentration in water (110 M) compensates for this low sensitivity, which is not true for low concentration and limited natural abundance nuclei, such as ^13^C, which are required to investigate metabolic substrate selection. Despite these sensitivity limitations, numerous studies have investigated cardiac metabolism with ^13^C-MRS in the isolated perfused rat heart [13]. To overcome the very low natural abundance of ^13^C (~1%) the perfused heart has to be supplied with ^13^C-labelled substrates. However, due to the low sensitivity, the detection of myocardial ^13^C labelled substrates *in vivo* using traditional MR methods remains extremely challenging. The process of hyperpolarization overcomes this insensitivity by transiently but dramatically increasing the signal available from a given ^13^C-labelled substrate. In this way, hyperpolarized magnetic resonance enables unprecedented visualization of normal and abnormal metabolism, allowing real-time measurement of instantaneous substrate uptake and enzymatic transformation *in vivo*[14,15].

This review focuses mainly on the changes in myocardial substrate selection and downstream metabolism of hyperpolarized ^13^C labelled pyruvate that have been shown in animal models of heart disease and details how these could translate into clinical practice with the recent arrival of sterile polarizer systems [16].

### Hyperpolarized techniques

The basis of magnetic resonance imaging lies in the interaction between the static magnetic field of the MRI system and the molecules of the body. When placed in the magnetic field, the molecules act like small bar magnets, aligning themselves in one of two orientations, either in the same direction as the field or opposed to it. The signal generated by the MRI system is then proportional to the difference in the number of molecules aligned in the two orientations, referred to as the polarization. At normal clinical magnetic field strengths and room temperature, the polarization is very small (e.g. at 3 T, the polarization is in the order of 0.001%). The aim of hyperpolarization techniques is to artificially increase the number of molecules in one orientation and thus the polarization level. This then results in an increase in the signal that can be generated by a given sample. Currently, there are four main approaches used to generate hyperpolarized compounds. These are brute force polarization [17], optical pumping of noble gases [18], parahydrogen-induced polarization (PHIP) [19] and finally, dynamic nuclear polarization (DNP), which will form the focus of the rest of this article [20].

#### Dynamic nuclear polarization

The DNP technique enhances the polarization of a specific nucleus (typically ^13^C or ^15^ N) within a particular molecule of interest [20]. It requires the mixing of the molecule to be hyperpolarized with a source of free electrons (radical). The mixed sample is then placed in a high magnetic field (typically 3.35 - 5 T) and rapidly frozen in liquid helium, reducing the sample temperature to approximately 1 K. In these conditions, the free electrons are nearly 100% polarized, whereas the nuclear spins of the sample molecule are still relatively poorly polarized. The high electron polarization is then transferred to the nuclear spins through the irradiation of the sample with microwave energy at a specific frequency, which is determined by the magnetic field strength and the atomic properties of the nuclei and radical within a given sample.

**Figure 1:**
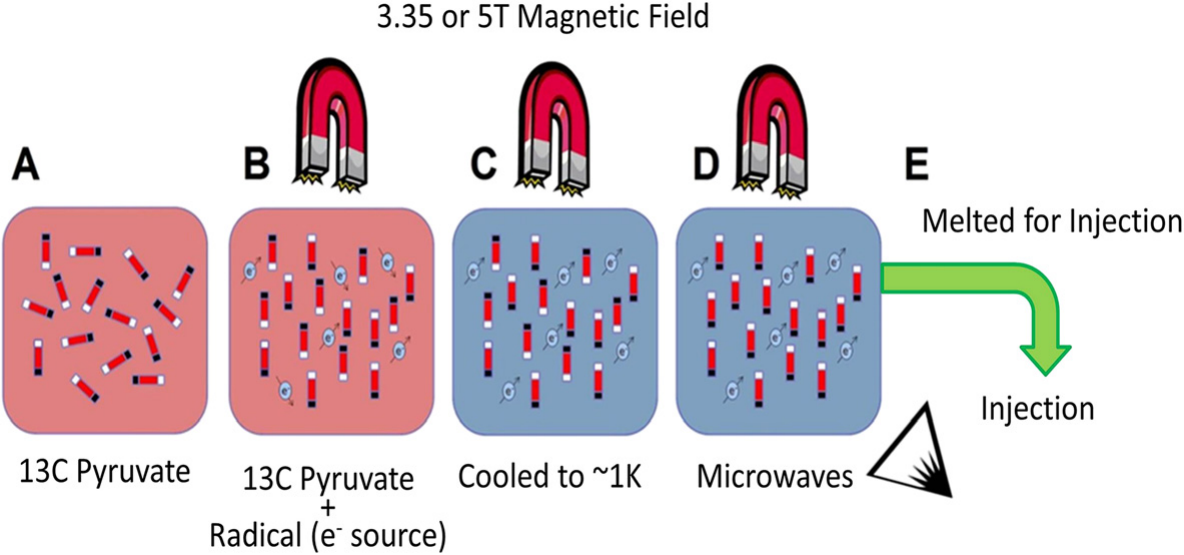
**The DNP process. (A)** Tracer Sample (^13^C pyruvate in this example) is placed in a strong magnetic field with a radical source of electrons **(B)**. The sample is cooled to very low temperatures **(C)** resulting in high electron polarization. Microwaves are used to transfer the spin polarization from electrons to the tracer **(D)**. The tracer is rapidly melted for injection **(E)**.

Despite the high level of polarization that can be achieved, the biological application of the DNP process has been limited as the hyperpolarization process needs to take place in the solid state. This limitation was removed by the recent development of the dissolution DNP process [20], where the highly polarized solid sample is rapidly melted with a bolus of superheated liquid. This generates an injectable sample, which retains a large proportion of the enhanced polarization and can be used as an *in vivo* MR contrast agent [20] (Figure 1).

Using this process DNP can increase the *in vivo* sensitivity of MRS to detect metabolic tracers more than 10,000-fold [20]. The enhanced signal then gradually returns back to the normal equilibrium over a period of time determined by the properties of the sample under investigation, typically on the order of 1–2 minutes. Thus, for the first time, high-resolution, highly reproducible metabolic assessment of substrate utilization in the heart by ^13^C-MRS and ^13^C cardiovascular magnetic resonance (CMR) has become possible [21].

### Hyperpolarized ^13^C pyruvate studies

**Figure 2:**
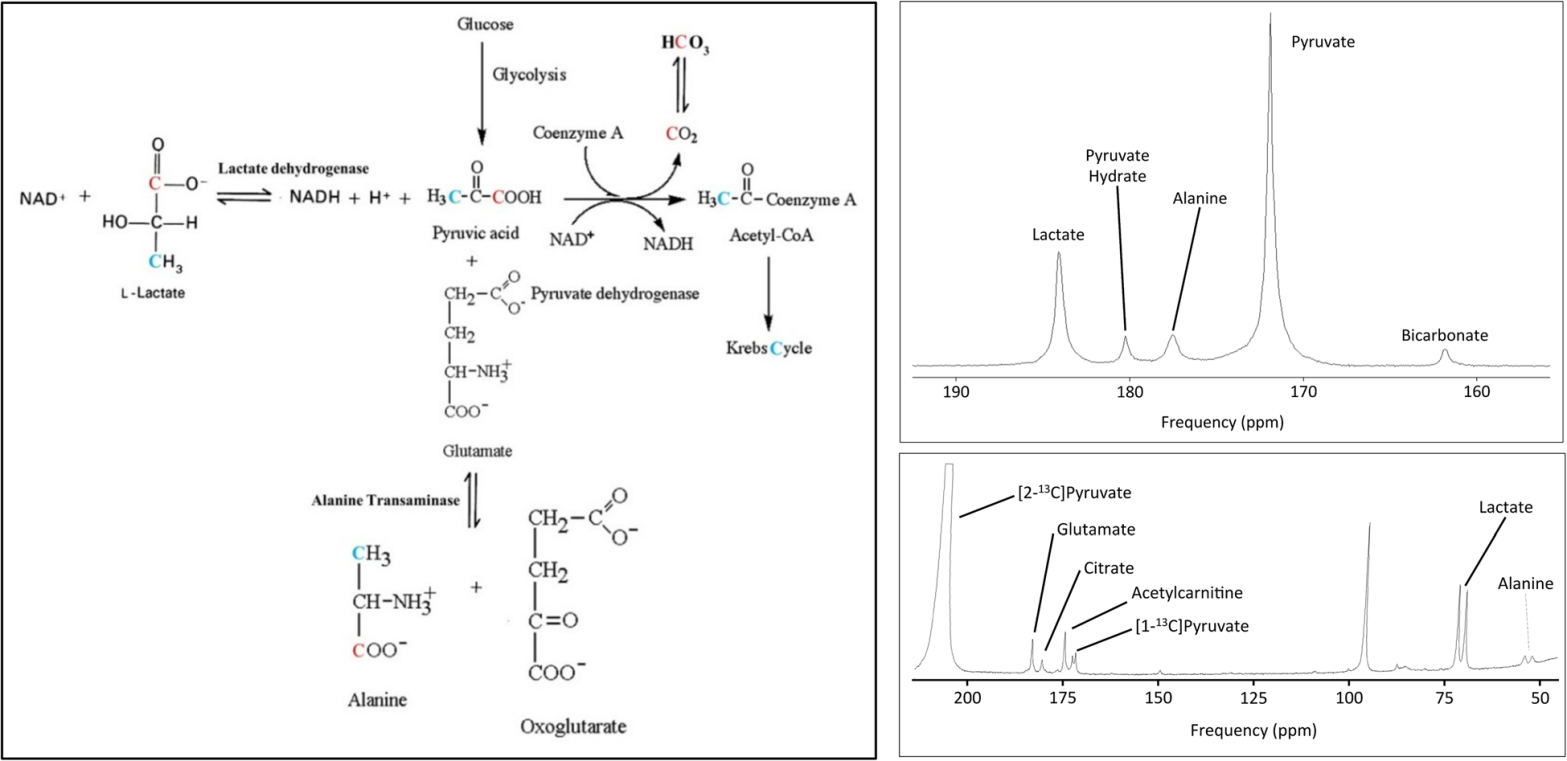
**Metabolic pathways interrogated according to **^**13**^**C labelled position, blue C2 position, red C1 position. (A)** [1-^13^C]pyruvate spectrum showing conversion to lactate, pyruvate hydrate, alanine and bicarbonate and **(B)** Example spectra acquired in the first 60s following [2-^13^C]pyruvate infusion in the in vivo rat heart. [2-^13^C]pyruvate is observed at 207.8 ppm. Peaks from (1) [5-^13^C]glutamate, (2) [1-^13^C]citrate, (3) [1-^13^C]acetylcarnitine, (4) [1-^13^C]pyruvate, (5) [2-^13^C]lactate & (6) [2-^13^C]alanine can be seen.

The majority of cardiac DNP investigations to date have used ^13^C-pyruvate to interrogate myocardial metabolism. The rationale for this lies in the fact that pyruvate sits at a key intersection of multiple metabolic pathways and plays an integral part in cellular energy homeostasis. For hyperpolarized ^13^C studies of cardiac metabolism, pyruvate has been labelled in the one-carbon [1-^13^C] and two-carbon [2-^13^C] positions. Depending on which carbon position is labelled with ^13^C, interrogation of different metabolic pathways can be achieved (Figure 2). As the fate of pyruvate; namely conversion to alanine (via Alanine Aminotransferase, ALT), lactate (via Lactate Dehydrogenase, LDH) and acetyl-CoA/CO_2_ (via Pyruvate Dehydrogenase, PDH), is dependent on prevailing metabolic conditions, this provides a window on several important metabolic processes that are essential to cardiac function, and which vary during differing disease processes. If pyruvate is ^13^C labeled in the third carbon position ([3-^13^C]-pyruvate) the methyl carbon group results in a T_1_ relaxation time that is short, making it an unattractive target for *in vivo* hyperpolarization studies. In contrast, the T_1_ relaxation times of [1-^13^C] and [2-^13^C]-pyruvate are sufficiently long to make them good targets for hyperpolarization.

### Hyperpolarized [1-^13^C]pyruvate studies

The initial hyperpolarized ^13^C MRS measurements of *in vivo* substrate selection were validated against analogous data collected *in vitro* and *ex vivo*. It was first demonstrated in the isolated perfused rat heart that infusion of hyperpolarized [1-^13^C]pyruvate, and MRS detection of total carbonic acid (^13^CO_2_ plus ^13^C-bicarbonate), measured flux through the PDH enzyme complex [22]. Subsequent *in vivo* work rapidly showed that if hyperpolarized [1-^13^C]Pyruvate was metabolized, the resulting signal was transferred to lactate, CO_2,_ bicarbonate and alanine allowing the assessment of 3 separate enzyme reactions, namely 1) LDH flux (pyruvate-lactate conversion), 2) PDH flux (pyruvate-CO_2_/bicarbonate conversion) and 3) ALT flux (pyruvate-alanine conversion, Figure 2) [15,23,24].

In disease models, alterations in the flux of hyperpolarized [1-^13^C]pyruvate through myocardial pyruvate dehydrogenase (PDH), as assessed by the production of ^13^C-bicarbonate, has not only been shown to be 65% lower than normal in the type 1diabetic heart but also to correlate with disease severity [15]. In addition, hyperpolarized [1-^13^C]pyruvate spectroscopy has been performed in models of cardiac hypertrophy allowing a greater understanding of the variation in substrate switching that occurs in different models. For example, in contrast to the spontaneously hypertensive rat, where a move away from predominantly fatty acid oxidative metabolism towards an increased reliance on glucose oxidation has been observed [25], left ventricular hypertrophy in the setting of hyperthyroidism was shown to be related to a reduced PDH flux [26]. Hyperpolarized [1-^13^C]pyruvate spectroscopy was also able to demonstrate that this inhibition of glucose oxidation in the hyperthyroid heart was mediated by Pyruvate Dehydrogenase Kinase (PDK). Treatment with dichloroacetic acid (DCA), a potent inhibitor of PDK, restored the metabolic flexibility of the hyperthyroid heart and the level of cardiac hypertrophy was significantly reduced [26]. The ability of hyperpolarized [1-^13^C] pyruvate spectroscopy to discriminate distinct patterns of metabolic dysregulation in different causes of cardiac hypertrophy is unparalleled and, therefore, has the potential to allow targeted therapeutics to prevent/treat different aetiologies of cardiac hypertrophy.

Myocardial oxygen consumption has been shown to be sensitive to cardiac substrate selection, with fatty acid utilization increasing oxygen consumption [27]. Whilst this is considered to be unimportant to physiology in the heart under conditions of normal oxygen supply, in the setting of ischemia or ischemia–reperfusion, increased metabolism of fatty acids impairs contractility and recovery [28] and multiple studies have proved that increased oxidation of carbohydrates relative to fatty acids improves the outcome after myocardial ischemia [29,30].

The effects of transient global ischaemia (10mins) followed by reperfusion on cardiac substrate selection have been investigated in a perfused heart model using hyperpolarized [1-^13^C]pyruvate. Using this method, it has been shown that in the early reperfusion period PDH flux was essentially zero and coupled with an increased appearance of [1-^13^C]lactate (via cytosolic LDH). This was followed later, within 20 minutes of reperfusion, by recovery of PDH flux observed through the reappearance of the products of PDH, ^13^CO_2_ and ^13^C-bicarbonate [31].

In addition to these spectral data acquisitions, pre-clinical experiments in both rodents and pigs have also demonstrated that metabolic maps of the spatial distribution of the downstream metabolites of hyperpolarized [1-^13^C]pyruvate; namely bicarbonate, lactate and alanine, can provide a sensitive marker of ischaemia, with myocardial lactate production during coronary occlusion providing a direct visualisation of ischaemia [24,32,33]. When oxygen is present, pyruvate is converted to acetyl-CoA, by Pyruvate Dehydrogenase (PDH), supplying substrate for the tricarboxylic acid (TCA) cycle. However, under anaerobic conditions, pyruvate is converted to lactate (via Lactate Dehydrogenase) with resulting NAD + production that allows glycolysis to continue to produce ATP in the absence of oxygen (the usual terminal electron acceptor during mitochondrial oxidative metabolism).

As current clinical imaging techniques rely on indirect measures of ischaemia (either perfusion abnormalities or changes in wall motion during stress), it is hoped that direct visual assessment of ischaemia in the form of lactate imaging will aid guided revascularisation. Given the evidence that only if revascularisation is targeted to the presence of significant myocardial ischaemic burden (>10%) [34] does it improve outcome, this potential for downstream metabolites of hyperpolarized [1-^13^C]pyruvate imaging to localise and grade the extent of myocardial ischaemia is potentially of great clinical importance.

Identifying areas of myocardium that are hibernating and would benefit from revascularization, and discriminating them from areas that are non-viable and would not recover after revascularization is another clinically important question to which hyperpolarized [1-^13^C]pyruvate imaging may be able to contribute. Hibernating myocardium is in essence a state of persistently impaired myocardial function at rest due to chronically reduced coronary blood flow, which can be partially or completely restored to normal either by improving blood flow or by reducing oxygen demand [35]. In viable myocardium, cell membrane integrity is typically retained, and there is some mitochondrial activity, together with an active glucose metabolism, existence of coronary flow, and the presence of contractile reserve [36]. As imaging of hyperpolarized [1-^13^C]pyruvate metabolism can produce localised metabolite maps of lactate, alanine and bicarbonate, the viability of myocardial segments could be evaluated on the basis of these datasets [24].

**Figure 3:**
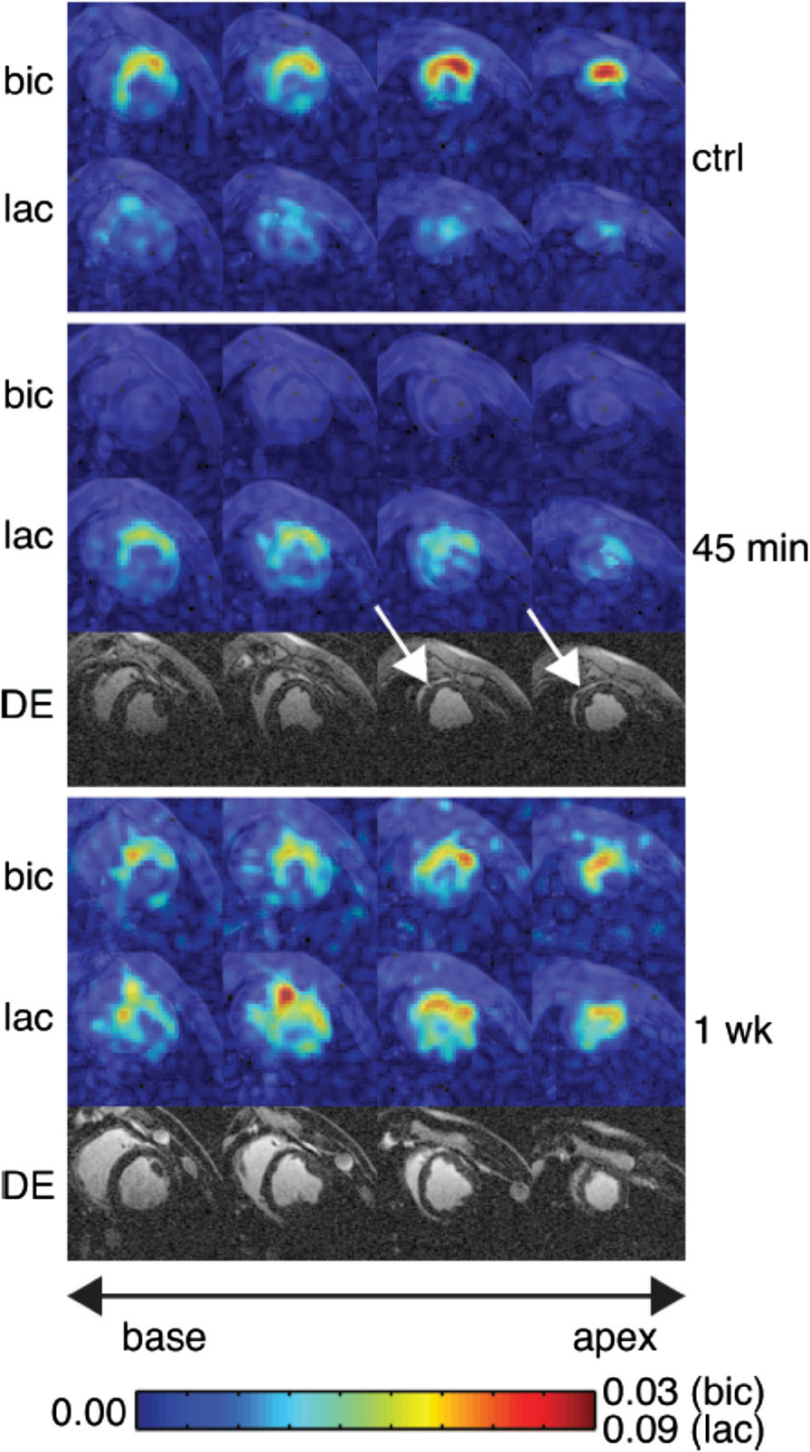
**Dual-gated short axis images in an animal exhibiting infarcted myocardium following 60-min LAD occlusion.** Images are shown at baseline, 45-min post-reperfusion, and 1-week post-reperfusion of the occluded artery. The color scale represents the image intensity, normalized by the maximum LV pyruvate signal intensity. Delayed enhancement revealed an enhancing anteroseptal infarct near the apex (arrows). Anteroseptal akinesis was present at the 45-min time point, persisting at 1 week. Apparent PDH flux in the bicarbonate images was reduced at 45 min, remaining suppressed at 1 week. A defect in myocardial lactate signal was observed in the infarct region (arrows), with elevated lactate in the peri-infarct region. (Reproduced with permission Magn Reson Med. 2013 Apr;69(4 ):1063–71).

Using an interleaved-frequency, time-resolved volumetric pulse sequence, robust and reliable three-dimensional measurements of cardiac metabolic signals have been obtained (Figure 3) [33,37]. These “single-shot” pulse sequences selectively produce images of metabolites in a very rapid time frame (~100 milliseconds per image). In large animal models of ischaemia-reperfusion, transient coronary occlusion resulted in regional hypokinesia with a reduced bicarbonate signal and an increased lactate signal consistent with acute infarction [33]. However, restoration of flow at 45 minutes was accompanied by restoration of function at 1 week and increased bicarbonate signal. This pattern of metabolites with normalisation of the bicarbonate signal, in the absence of late gadolinium enhancement, is consistent with viable myocardium. However, in a perfused heart model of chronic infarction, using ^13^C-hyperpolarized metabolite maps, the combination of reduced perfusion and significant reductions in both bicarbonate and lactate signals after prolonged coronary artery occlusion has been shown to reflect a loss of normal glycolytic metabolism indicative of cell membrane integrity disruption and non-viability [32].

In the majority of cases suitability for coronary revascularisation in the presence of depressed myocardial systolic function is based on the detection of regional myocardial viability as assessed by myocardial perfusion scanning (MPS) and stress echocardiography. However, recent results from the STICH trial, albeit using global measures of viability, did not show an advantage to viability assessment pre coronary artery bypass grafting, disrupting these traditionally accepted methods of assessing suitability for revascularisation [38]. It is now clear that better detection of viable myocardium is needed and the ability of ^13^C hyperpolarized imaging to detect and localise specific patterns of myocardial metabolism associated with ischaemia and viability promises to be an exciting advance in this area of cardiac imaging.

#### Intracellular pH assessment

The rapid onset of acidosis is another well-documented characteristic of myocardial ischaemia [39,40]. Under poor coronary perfusion, increased anaerobic glycolysis produces intracellular protons and lactic acid that accumulate in the intra- and extracellular spaces [41] and decrease intracellular pH (pH_i_) [42]. Although transient acidosis during ischaemia may be beneficial as it decreases contractility and conserves ATP for ion transport [43], the ATP reduction caused by severe and sustained ischaemia decreases Na^+^/K^+^-ATPase activity, which increases myocardial Na^+^ levels. This in-turn inhibits Ca^2+^ extrusion via the Na^+^/Ca^2+^ exchanger, elevating myocardial Ca^2+^ and damaging the myocardium [44].

^31^P-MRS has long been the gold standard for pH_i_ measurement in the isolated perfused heart, based on the chemical shift of the inorganic phosphate (P_i_) peak [45]. However, ^31^P MRS cannot measure cardiac pH_i_*in vivo*, because 2,3-diphosphoglycerate (2,3-DPG) in the ventricular blood contaminates the myocardial P_i_ peak. Recently, the pH-dependent equilibrium between bicarbonate and CO_2_ has been used to measure extracellular pH (pH_o_) non-invasively in tumours [14]. By infusing hyperpolarized ^13^C-bicarbonate intravenously, magnetic resonance has been used to image the distribution of hyperpolarized bicarbonate and CO_2_ and a pH map generated using the Henderson–Hasselbalch equation:

$$\mathrm{pH}=pK_{a}+\log\left(\frac{\left[\mathrm{HC}O_{3}^{-}\right]}{\left[CO_{2}\right]}\right)$$

As infusion of hyperpolarized [1-^13^C]pyruvate results in mitochondrial production of hyperpolarized ^13^CO_2_ by pyruvate dehydrogenase, which itself is in equilibrium with [^13^C]bicarbonate (due to the action of carbonic anhydrase), a similar approach has been used for measuring pH_i_ in both the perfused and *in vivo* rat heart [23]. Using hyperpolarized [1-^13^C]pyruvate spectroscopy in the perfused heart it was demonstrated that the H^13^CO_3_^-^/^13^CO_2_ ratio offered an accurate method to measure cardiac pH_i_ before and immediately after ischaemia [23]. As severe acidosis has been linked to myocardial cell death, this non-invasive assessment of myocardial pH_i_ after myocardial infarction may prove a useful prognostic marker of recovery.

### Hyperpolarized [2-^13^C]pyruvate studies

If pyruvate is enriched with ^13^C on the second carbon atom, the hyperpolarized label is not lost in the cleavage of pyruvate into acetyl-CoA and carbon dioxide (^13^CO_2_). Instead the ^13^C label is carried through acetyl-CoA and into the TCA cycle (Figure 2) allowing for the observation of various TCA cycle intermediates in real-time [46]. This has allowed TCA flux to be investigated in multiple cardiac disease models.

**Figure 4:**
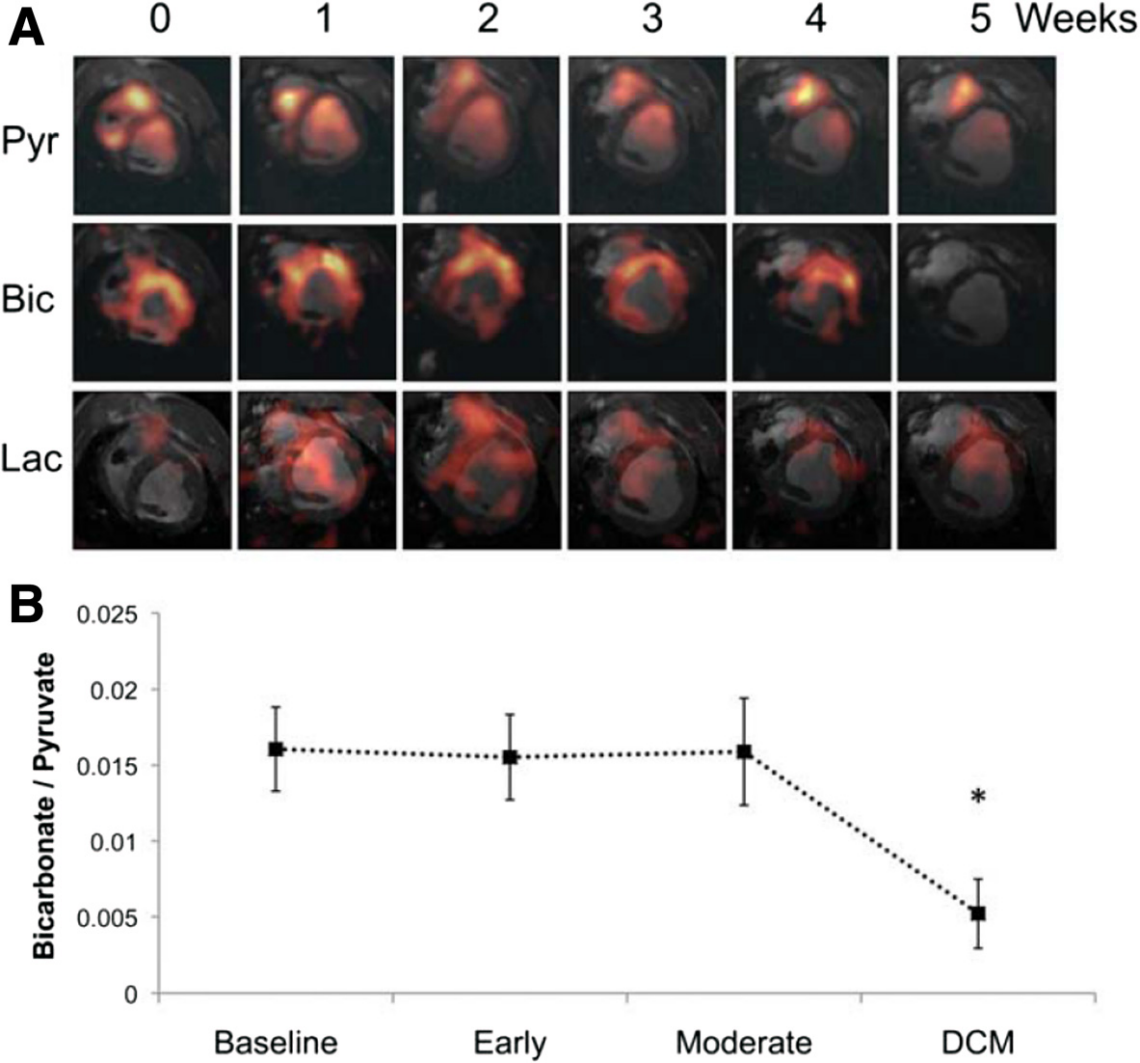
**Hyperpolarized [1-**^**13**^**C]pyruvate CMR showing alterations to pyruvate dehydrogenase complex (PDC) flux and [**^**13**^**C]lactate production with the pathogenesis of dilated cardiomyopathy (DCM). (A)** Representative pyruvate (Pyr, top), bicarbonate (Bic, middle), and lactate (Lac, bottom) ^13^C CMR images taken from the same pig and at weekly intervals during the pacing protocol, until DCM developed. The images displayed for each metabolite were selected from the same, mid-papillary slice and in the same respiratory cycle. Signal intensity in the pyruvate image was scaled based on 15–100% of the maximum pyruvate signal at week 0, whereas the bicarbonate and lactate signal intensities were scaled based on 15–100% of the maximum bicarbonate signal intensity at week 0. **(B)** Relative changes to PDC flux with DCM in five pigs.(Reproduced with permission Eur J Heart Fail. 2013 February; 15(2): 130–140).

The use of hyperpolarized CMR at multiple time-points following a myocardial infarction induced by ligation of the left anterior descending coronary artery has shown, *in vivo*, that tricarboxylic acid (TCA) cycle flux (as indicated by the reduced production of citrate and glutamate from [2-^13^C]pyruvate) is significantly reduced from six weeks after infarction in the infarcted heart [47]. Also, using metabolic mapping in a pacing induced porcine model of dilated cardiomyopathy (DCM) it has been shown that, despite early impairment of cardiac energetics (reduced PCr/ATP ratio) and changes in [2-^13^C]pyruvate incorporation into the TCA cycle (reduced ^13^C glutamate production), pyruvate oxidation was maintained until overt DCM developed, when the heart’s capacity to oxidize both pyruvate and fats was reduced (Figure 4) [48]. As a result, hyperpolarized [2-^13^C]pyruvate imaging may be important to characterize metabolic changes that occur during heart failure progression and provide potential treatment targets.

#### Translation of pyruvate studies to humans

These pre-clinical cardiac results, combined with studies using hyperpolarized magnetic resonance in oncology [49], have led to the granting of an “Investigational New Drug” approval for hyperpolarized pyruvate from the FDA and the first application of DNP hyperpolarized magnetic resonance in humans at the University of California in San Francisco [50]. In these “first in man” studies, the potential for hyperpolarized magnetic resonance to stage prostate cancer has been investigated. Initial results indicate that the measurement of a significant lactate signal following administration of hyperpolarized pyruvate provides a sensitive marker of malignant versus benign tissue [49]. Whilst the accumulation of lactate in tumours is well known, hyperpolarized magnetic resonance is the only technique to offer the clinical potential to be able to assess the level of lactate non-invasively in humans. The recent production, by GE Healthcare, of a sterile “SpinLab” hyperpolarizer system [16] means that the translation of cardiac ^13^C pyruvate studies from animal models to humans is imminent [51].

### Safety and tolerability of pyruvate

Pyruvate itself has been reported as an attractive treatment for heart failure and has been the subject of multiple clinical studies [52-54]. Supra-physiological levels of pyruvate (150 mmol/L infused at up to 740 ml/hr) have been infused into the coronary arteries invasively at angiography and have been shown in small studies to be well tolerated and result in increased cardiac output, decreased pulmonary capillary-wedge pressure and decreased heart rate in patients with dilated cardiomyopathy and heart failure [54]. Despite the safety and tolerability of slow infusions in the above heart failure study and high doses in chronic liver disease studies (up to 82.4 g/day for 10 days) [55], owing to the rapid loss of hyperpolarization and thus the short imaging window it will provide, pyruvate injection must be administered as a bolus injection at a rate of ~ 5 ml/second, resulting in potentiality supra-physiological doses.

The first application of hyperpolarized magnetic resonance in humans (using bolus injection) has now been performed at the University of California in San Francisco (UCSF) [56,57]. In this “first in man” study, the potential for hyperpolarized magnetic resonance to stage prostate cancer and assess response to treatment has been investigated. Although no studies of human cardiac metabolism have been performed to date, the pyruvate concentrations used in the UCSF clinical trial of prostate cancer are likely to be identical to initial cardiac studies.

### Other metabolic tracers

In order to be a useful metabolic probe, any potential tracer molecule needs to have the following physical properties; 1) the tracer needs to maintain its polarization (i.e. have a sufficiently long T_1_ relaxation time) for a period of time necessary for the study to be carried out, 2) the compound needs to be enriched with non-zero nuclear spin nuclei, 3) when the mixture is frozen it needs to form a glass rather than a crystallized solid (as successful polarization levels are generally achieved by DNP with glass formation), and finally 4) that the tracer is rapidly incorporated into a metabolic pathway.

In addition to pyruvate and bicarbonate, several other potential hyperpolarized probes have been proposed. For example, distinct regions of the TCA cycle have been assessed using hyperpolarized [1-^13^C]glutamate and [1,4-^13^C_2_]fumarate, the respective conversions of which to [1-^13^C]α-ketoglutarate and [1,4-^13^C_2_]malate have been demonstrated *in vivo*[58,59]. Hyperpolarized butyrate has been used as a marker of short chain fatty acid metabolism [60] and hyperpolarized [1-^13^C]acetate has also been used in preclinical models as an assay for intracellular CoA levels [61]. Hyperpolarized glucose, vitamin C, lactate and alanine, amongst many others, have also been demonstrated to be useful *in vivo*[62-66]. However, the extent to which these, or any other, tracers can be used in clinical applications has yet to be determined.

### Other potential cardiac applications

#### Angiography

The large signal-to-noise (SNR) achievable with hyperpolarized ^13^C-labeled tracer molecules, combined with the low background signal results in a high contrast-to-noise ratio (CNR), which is ideal for angiographic examination [67]. Therefore, the potential to use hyperpolarized agents for angiography has also been explored [68,69], focusing mainly on coronary [70] and pulmonary [71] artery imaging. However, due to the fact that the gyromagnetic ratio of ^13^C is a quarter that of ^1^H the large demands placed on the imaging gradients necessitates powerful gradient amplifiers and, when combined with rapid imaging techniques, is likely to limit the achievable spatial resolution.

#### Perfusion imaging

Routine CMR assessments of myocardial perfusion are generally based on the first passage of a gadolinium based contrast agent [72]. However, gadolinium perfusion is an indirect assessment of perfusion which makes absolute quantification of myocardial perfusion difficult [73]. This problem may be overcome by hyperpolarized perfusion measurements, which rely directly on the signal obtained from the hyperpolarized tracer and so allow for absolute quantification. Although measurements have been made in porcine models [74], the continual decay of the hyperpolarized signal needs to be accounted for and represents a limitation of the technique [75].

## Conclusions

Hyperpolarization results in a substantially increased signal which overcomes the sensitivity limitations of some multi-nuclear CMR applications. When combined with the metabolic tracers [1-^13^C] and [2-^13^C] pyruvate, this has resulted in unparalleled real time imaging of myocardial substrate metabolism *in vivo*. With imminent translation into human studies this novel technique has the potential to provide important and clinically useful information in the setting of multiple cardiac diseases including ischemic heart disease, cardiac hypertrophy and heart failure. There is clear potential for hyperpolarized imaging to have a significant impact in the future of CMR as a unique metabolic imaging modality.

## Competing interests

The authors declare that they have no competing interests.

## Authors’ contributions

OR & DT performed the literature search OR drafted the manuscript. Both authors read and approved the final manuscript.
